# Клиническая, гормональная и молекулярно-генетическая характеристика случаев нарушения формирования пола при кариотипе 46,XY, ассоциированных с вариантами в гене <i>HSD17B3</i>

**DOI:** 10.14341/probl13415

**Published:** 2024-01-09

**Authors:** Н. Ю. Калинченко, Н. А. Макрецкая, А. А. Колодкина, В. А. Иоутси, В. М. Петров, А. Н. Тюльпаков

**Affiliations:** Национальный медицинский исследовательский центр эндокринологии; Медико-генетический научный центр имени академика Н.П. Бочкова; Национальный медицинский исследовательский центр эндокринологии; Национальный медицинский исследовательский центр эндокринологии; Национальный медицинский исследовательский центр эндокринологии; Медико-генетический научный центр имени академика Н.П. Бочкова; Российская детская клиническая больница

**Keywords:** нарушение формирования пола, недостаточность 17-гидроксистероиддегидрогеназы типа 3, HSD17B3, 46, XY, тестостерон, андростендион

## Abstract

ОБОСНОВАНИЕ. Недостаточность 17β-гидроксистероиддегидрогеназы типа 3 (HSD17B3) является редким вариантом нарушения формирования пола (НФП) при кариотипе 46,XY. В настоящее время в отечественной литературе отсутствуют обобщенные данные по указанной группе пациентов, что затрудняет проведение своевременной диагностики и выбор тактики лечения.ЦЕЛЬ. Дать клиническую, гормональную и молекулярно-генетическую характеристику случаев НФП 46,XY, ассоциированных с вариантами в гене HSD17B3.МАТЕРИАЛЫ И МЕТОДЫ. Одноцентровое ретроспективное исследование, включившее 310 пациентов с НФП 46,XY. Пациентам проводилось комплексное обследование, включающее исследование стероидного профиля методом высокоэффективной жидкостной хроматографии с тандемным масс-спектрометрическим детектированием, а также молекулярно-генетическое исследование с использованием NGS.РЕЗУЛЬТАТЫ. По результатам проведенных молекулярно-генетических исследований биаллельные нуклеотидные замены в гене HSD17B3 выявлены в 13 случаях, что составило 4,2% от общего числа пациентов с НФП 46,XY. Все 13 пациентов с биаллельными вариантами в гене HSD17B3 зарегистрированы в женском поле. Соотношение концентраций андростендиона/тестостерона в крови в этой группе варьировало от 1,4 до 8,9. Два варианта в гене HSD17B3 встречались у нескольких пациентов: c.277+4A>T (на 6 хромосомах) и c.729_735del:p.V243fs (на 9 хромосомах). Выявлено 4 ранее не описанных варианта. Моноаллельные нуклеотидные замены в гене HSD17B3 выявлены в 7 случаях, что составило 2,3% от общего числа пациентов с НФП 46,XY. Строение наружных гениталий в данной группе соответствовало стадиям 3–4 по Прадеру. У 1 пациента в гене HSD17B3 выявлен патогенный вариант c.277+4A>T, в остальных случаях определялись варианты с неопределенным клиническим значением.ЗАКЛЮЧЕНИЕ. В структуре НФП 46,XY пациенты с биаллельными вариантами в гене HSD17B3 выявлены в 4,2% случаев, с моноаллельными вариантами — в 2,3% случаев. Обнаружены 4 ранее не описанные варианта в гене HSD17B3.

## Abstract (en)

BACKGROUND: Deficiency of 17β-hydroxysteroid dehydrogenase type 3 (HSD17B3) is a rare variant of 46,XY disorders of sex development (DSD).AIM: To give clinical, hormonal and molecular genetic characteristics of cases of 46,XY DSD associated with variants in the HSD17B3 gene.MATERIALS AND METHODS: The study included 310 patients with 46,XY DSD for the period from 2015 to 2019. The patients underwent a comprehensive examination, including a study of the steroid profile by high-performance liquid chromatography with tandem mass spectrometric detection, as well as a molecular genetic analysis using NGS.RESULTS: According to the results of molecular genetic studies, biallelic nucleotide substitutions in the HSD17B3 gene were detected in 13 cases, which accounted for 4.2% of the total number of patients with 46,XY DSD. All 13 patients with biallelic variants in the HSD17B3 gene were registered as females. The ratio of androstenedione/testosterone concentrations in the blood in this group ranged from 1.4 to 8.9. 2 variants in the HSD17B3 gene were found in several patients: c.277+4A>T (on 6 chromosomes) and c.729_735del:p.V243fs (on 9 chromosomes). 4 novel variants have been identified. Monoallelic nucleotide substitutions in the HSD17B3 gene were detected in 7 cases, which accounted for 2.3% of the total number of patients with 46,XY DSD. External genitalia in this group corresponded to Prader stages 3–4. In 1 patient, a pathogenic variant c.277+4A>T was detected in the HSD17B3 gene, in other cases variants with uncertain significance were detected.CONCLUSION: In the structure of 46,XY DSD, patients with biallelic variants in the HSD17B3 gene were identified in 4.2% of cases, with monoallelic variants — in 2.3% of cases. 4 novel variants were found in the HSD17B3 gene.

## ОБОСНОВАНИЕ

HSD17B3 (17β-гидроксистероиддегидрогеназа типа 3) — это микросомальный фермент стероидогенных клеток яичек, катализирующий этап превращения слабых андрогенов андростендиона (А) и 5α-андростандиона в их функционально активные метаболиты тестостерон (Т) и дигидротестостерон (ДГТ) соответственно [1]. Снижение активности фермента в период внутриутробного развития плода с кариотипом 46,XY приводит к дефициту активных андрогенов и, как результат, нарушению маскулинизации наружных половых органов при нормально развитых яичках и отсутствии дериватов Мюллеровых протоков. В связи с тем, что при рождении у пациентов с дефицитом HSD17B3 строение наружных половых органов (НПО) ближе к женскому, у них нередко устанавливается женский пол воспитания [2]. Однако в период полового созревания активируются другие изоферменты 17-β-гидроксистероиддегидрогеназы, приводящие к значительному повышению уровня активных андрогенов и, как следствие, маскулинизации пациенток с женским паспортным полом.

Длительное время считалось, что недостаточность HSD17B3 является крайне редким вариантом нарушения стероидогенеза [3]. Однако с внедрением в практику методов молекулярно-генетической диагностики, позволивших проведение когортных исследований, оказалось, что до 4% случаев нарушения формирования пола (НФП) 46,XY составляет дефицит HSD17B3 [4]. Более того, в регионах, где распространены близкородственные браки, частота встречаемости дефицита HSD17B3 может достигать 1:300–100 новорожденных мальчиков [5][6].

Дефицит HSD17B3 прежде всего необходимо дифференцировать с резистентностью к андрогенам (синдром тестикулярной феминизации) и с дефицитом 5 α-редуктазы, при которых также будут отмечаться почти правильное женское строение НПО в сочетании с правильно сформированными яичками, высоким уровнем АМГ и отсутствием дериватов Мюллеровых протоков. При общности клинических проявлений подходы к лечению данных заболеваний могут значительно отличаться. В связи с чем своевременная диагностика дефицита HSD17B3 является актуальной для определения правильной тактики ведения пациента.

Ранее нами было представлено два первых в России случая молекулярно-генетической верификации дефицита HSD17В3 [7]. В настоящей публикации мы приводим данные обследования 13 новых случаев дефицита HSD17В3, обусловленных биаллельными мутациями в гене HSD17В3. Помимо этого, интересной находкой, на наш взгляд, является обнаружение у части пациентов с НФП 46,XY гетерозиготных вариантов в гене HSD17В3, что позволяет предположить возможную роль таких дефектов в олигогенной природе заболевания.

## ЦЕЛЬ ИССЛЕДОВАНИЯ

Дать клиническую, гормональную и молекулярно-генетическую характеристику случаев НФП 46,XY, ассоциированных с вариантами в гене HSD17B3.

## МАТЕРИАЛЫ И МЕТОДЫ

Место проведения. ФГБУ «НМИЦ эндокринологии» Минздрава России.

Время исследования. Январь 2015–декабрь 2019 гг.

Анализ данных проведен на основании предоставленных выписок для проведения генетического исследования пациентов с НФП 46,XY и у пациентов, проходивших обследование в Институте детской эндокринологии ФГБУ «НМИЦ эндокринологии» Минздрава России за период с 2015 по 2019 гг.

## Изучаемые популяции (одна или несколько)

Популяция: пациенты от 0 до 17 лет с НФП 46,XY.

Критерии включения: несоответствие между хромосомным мужским полом 46,XY и строением наружных и/или внутренних половых органов.

Критерии исключения: наличие синдромальной патологии с множественными пороками развития.

## Способ формирования выборки из изучаемой популяции (или нескольких выборок из нескольких изучаемых популяций)

Сплошной способ формирования выборки.

## Дизайн исследования

Одноцентровое ретроспективное одновыборочное исследование, включившее 310 пациентов с НФП 46,XY.

## Методы

В исследование включались пациенты с НФП при кариотипе 46,XY. НФП определялось как несоответствие хромосомного пола фенотипическому полу. Оценка строения НПО осуществлялась по шкале Прадера. Из исследования исключались пациенты, у которых имелось сочетание НФП 46,XY с синдромальной патологией.

Пациентам проводилось комплексное обследование, включавшее: оценку строения наружных половых органов по Прадеру, ультразвуковое исследование (УЗИ) малого таза, паховых каналов, органов мошонки, исследование гормонального статуса — лютеинизирующий гормон (ЛГ), фолликулостимулирующий гормон (ФСГ), тестостерон, эстрадиол (Э2). У части пациентов проводилась проба с хорионическим гонадотропином, исследовался стероидный профиль методом высокоэффективной жидкостной хроматографии с тандемным масс-спектрометрическим детектированием, включавший 14 показателей: альдостерон, кортизон, кортизол, 21-дезоксикортизол, кортикостерон, 11-дезоксикортизол, андростендион, дезоксикортикостерон, тестостерон, 17-гидроксипрогестерон, 17-гидроксипрегненолон, дегидроэпиандростерон, прогестерон, прегненолон. Пробоподготовку образцов сыворотки крови, подготовку градуировочных образцов и образцов контроля качества проводили аналогично описанной ранее методике [8]. Для проведения анализа использовали хроматографическую систему Agilent 1290 Infinity II тандемный гибридный трехквадрупольный масс-спектрометр AB Sciex QTrap 5500. Детектирование проводили в режиме мониторинга множественных реакций, подбор параметров для этого режима осуществляли для каждого компонента индивидуально по образцам стандартных веществ.

Молекулярно-генетический анализ проводился в лаборатории отделения наследственных эндокринопатий. Геномную ДНК выделяли из лейкоцитов периферический крови стандартным методом (набор Pure Link, Genomic DNA Mini Kit, Life Technologies, США). Для молекулярно-генетического анализа применялся метод NGS. Использовалась разработанная в отделении наследственных эндокринопатий панель праймеров для мультиплексной ПЦР и секвенирования с применением технологии Ion Ampliseq™ Custom DNA Panel (Life Technologies, США). Панель праймеров «Нарушения формирования пола» охватывает кодирующие области следующих генов: AKR1C2, AKR1C4, AMH, AMHR2, AR, ARX, ATRX, CBX2, CYB5A, CYP11A1, CYP17A1, DHCR7, DHH, EMX2, ESR2, FGD1, FGF9, FGFR2, FKBP4, FOXF2, FOXL2, HOXA13, HSD17B3, HSD3B2, ICK, LHCGR, LHX1, LHX9, MAMLD1, MAP3K1, MID1, NR0B1, NR5A1, POR, PTGDS, SOX9, SRD5A2, SRY, STAR, SUPT3H, TSPYL1, WNT4, WT1, ZFPM2. Подготовка библиотек проводилась в соответствии с рекомендациями производителей. Секвенирование осуществлялось на полупроводниковом секвенаторе PGM (Ion Torrent, Thermo Scientific, США) или Illumina MiSeq (Illumina, США). Биоинформатическая обработка результатов секвенирования проводилась с помощью программных модулей Torrent Suite 4.2.1 (Ion Torrent, Life Technologies, США) или Genome Analysis ToolKit (GATK) ver. 4.1.2.0 (Broad Institute, Cambridge, MA, USA). Для аннотирования вариантов нуклеотидной последовательности использовался пакет программ ANNOVAR ver. 2018Apr16. После анализа полученных данных проводилось подтверждение полученных мутаций на секвенаторе Genetic Analyzer Model 3130 (Life Technologies, США). Оценка патогенности вариантов нуклеотидной последовательности проводилась согласно международным и российским рекомендациям [9][10]. Нумерация кодирующей последовательности гена дана по референсу NM_000197.1 (http://www.ncbi.nlm.nih.gov/genbank).

## Этическая экспертиза

Проведение исследования одобрено Локальным этическим комитетом ФГБУ «НМИЦ эндокринологии» Минздрава России 01.01.2021 г.

## РЕЗУЛЬТАТЫ

В исследование включено 310 пациентов с НФП 46,XY. По результатам проведенных молекулярно-генетических исследований, биаллельные нуклеотидные замены в гене HSD17B3 были выявлены в 13 случаях, что составило 4,2% от общего числа пациентов с НФП 46,XY. Моноаллельные нуклеотидные замены в гене HSD17B3 были выявлены в 7 случаях, что составило 2,3% от общего числа пациентов с НФП 46,XY.

## Пациенты с биаллельными вариантами в гене HSD17B3

Все 13 пациентов с биаллельными мутациями в гене HSD17B3 при рождении были зарегистрированы в женском паспортном поле. Анамнестические данные были доступны по 9 пациентам. Возраст на момент выявления НФП 46,XY варьировал от рождения до 16 лет. Поводом для обследования послужили выявление гонад в паховых областях, половых губах (у 7) или проявления маскулинизации в пубертатном периоде (у 6). У 4 пациентов первоначально был ошибочно диагностирован синдром резистентности к андрогенам (СРА), и у 1 — врожденная дисфункция коры надпочечников (ВДКН). Возраст верификации дефицита HSD17B3 варьировал от 1 года до 16 лет.

Результаты исследования А, Т и А/Т были доступны в 8 случаях. У 2 пациентов в возрасте 10 мес и 3,5 года обследование было проведено на фоне пробы с хорионическим гонадотропином человека (ХГЧ), и стимулированные уровни А, Т, и соотношение А/Т у них варьировали от 5,6 до 4,4 нмоль/л, 3,6 до 0,7 нмоль/л и от 1,4 до 6,3 соответственно. У 5 пациентов в возрасте от 11 до 16 лет на фоне пубертата оценивались только базальные показатели А, Т и А/Т, составившие 22–64,5 нмоль/л, 4,4–19 нмоль/л и 2,8–8,9 соответственно. У 1 пациентки в возрасте 3,5 года так же оценивались только базальные показатели А 0,9 нмоль/л и Т 0,3 нмоль/л, А/Т 3,0. Проба с ХГЧ не проводилась, учитывая наличие генетически верифицированного дефицита HSD17B3 до этапа гормонального обследования.

При молекулярно-генетическом обследовании у 7 пациентов были идентифицированы гомозиготные варианты, и у 6 — компаунд-гетерозиготные варианты в гене HSD17B3. Чаще других выявлялись две ранее описанных мутации: c.277+4A>T (на 6 хромосомах) и c.729_735del:p .V243fs (на 9 хромосомах). В ходе исследования было обнаружено 4 ранее не описанных варианта, 2 из которых были классифицированы как патогенные (c.673-2A>G, c.111_118delAGTTTTGC p.K37NfsX40) и 2 — как вероятно патогенные (c.598G>A:p .A200T, c.638A>C p.Q213P) [9][10] (табл. 1).

**Table:** Таблица 1. Клиническая, гормональная и молекулярно-генетическая характеристика пациентов с биаллельными вариантами в гене HSD17B3 Примечания: ВДКН — врожденная дисфункция коры надпочечников; СРА — синдром резистентности к андрогенам; Б — базально, пп — после пробы, А — андростендион, Т — тестостерон.

№	Фенотип при рождении/ диагностике заболевания	Возраст диагностики	Направляющий диагноз	А/Т базальные / после пробы с ХГЧ (нмоль/л)	Варианты в гене HSD17B3	Оценка патогенности для новых вариантов	HGMD_ID
1	Женский правильный, гонады в половых губах. Гонадэктомия в 1 год	3 года	СРА	N/A	c.397G>A:p .G133R c.729_735del:p .V243fs		CM1511850 [11] CD963074 [2]
2	Гипертрофированный клитор, в области паховых каналов образования по 1 мл, отверстие мочеиспускательного канала открывается над входом во влагалище	10 мес	ВДКН, лечение кортефом	б: 0,5/0,29 пп: 5,02/3,6	c.277+4A>T c.598G>A:p .A200T	LP (PM2, PP3, PM5, PM1, PM3, PP4)	CS002140 [12] Новый
3	Женский правильный, гонады в половых губах. Гонадэктомия в 1 год	N/A	СРА	N/A	c.673-2A>G c.729_735del:p .V243fs	P (PM2, PVS1, PM3, PP4)	Новый CD963074 [2]
4	Женский правильный, в паховых каналах двусторонние образования до 2 мл	N/A	СРА	N/A	c.527A>C:p .Q176P c.527A>C:p .Q176P		CM960845 [2]
5	Женский правильный, в три года паховые грыжи	3,5 года		пп: 4,4/0,7	c.277+4A>T c.729_735del:p .V243fs		CS002140 [12] CD963074 [2]
6	Женский правильный, в большой губе образование	1 год		б (3 года): 0,9/0,3	c.729_735del:p .V243fs c.729_735del:p .V243fs		CD963074 [2]
7	Женский правильный. С 13 лет гипертрофия клитора, барифония	13 лет		б: 45,7/12,7	c.203T>G:p .L68R c.203T>G:p .L68R		CM154897 [13]
8	Женский правильный, гонады в паховых каналах. С 11 лет гипертрофия клитора	13 лет		б: 29/5,1	c.638A>C:p .Q213P c.729_735del:p .V243fs	LP (PM2, PP3, PM1, PM3, PP4)	Новый CD963074 [2]
9	Женский правильный. С 11 лет гипертрофия клитора, в половых губах гонады	12,5 года		б: 54/19	c.111_118delAGTTTTGC:p .K37NfsX40 c.729_735del:p .V243fs	P (PM2, PVS1, PM3, PP4)	Новый CD963074 [2]
10	Женский правильный, гонады в половых губах. Гонадэктомия в 1 год	5 лет	СРА	N/A	c.277+4A>T c.277+4A>T		CS002140 [12]
11	Женский правильный. С 10 лет гипертрофия клитора, гонады в паховых каналах	12 лет		б: 22/4.4	c.277+4A>T c.277+4A>T		CS002140 [12]
12	Умеренно гипертрофия клитора, с 11 лет резкое увеличение, гонады в половых губах	16 лет		б: 67,4/7,6	c.160A>G:p .T54A c.160A>G:p .T54A		CM164516 [14]
13	Женский правильный. С 12 лет гипертрофия клитора	13 лет		N/A	c.729_735del:p .V243fs c.729_735del:p .V243fs		CD963074 [2]

## Пациенты с моноаллельными вариантами в гене HSD17B3

У 7 пациентов с промежностной формой гипоспадии и зарегистрированных в мужском паспортном поле была выявлена одна гетерозиготная мутация в гене HSD17B3, что составило 2,3% от общего числа пациентов с НФП 46,XY. У 1 пациента был выявлен описанный ранее патогенный вариант c.277+4A>T, который был также частой находкой у пациентов с биаллельными мутациями. В остальных случаях все моноаллельные мутации были расценены как варианты с неопределенной клинической значимостью (табл. 2). Среди последних заслуживает внимания вариант c.133C>T:p .R45W, который был обнаружен у 3 пациентов.

**Table:** Таблица 2. Клиническая и молекулярно-генетическая характеристика пациентов с моноаллельными вариантами в гене HSD17B3 Примечания: н/выявлено — других патогенных вариантных замен в анализируемых генах не выявлено, СФ 1 — стероидогенный фактор 1

№	Фенотип при рождении	Диагноз уточненный	Варианты в гене HSD17B3	HGMD_ID
1	Промежностная гипоспадия, Прадер 3, гонады в мошонке	Дефицит CФ 1	c.277+4A>T	CS002140 [12]
2	Промежностная гипоспадия, Прадер 4, гонады в мошонке	Дефицит CФ 1	c .C133T:p .R45W	CM1616127 [15]
3	Промежностная гипоспадия, Прадер 3, гонады в мошонке	Панель «НФП» — н/выявлено	c .C133T:p .R45W	CM1616127 [15]
4	Промежностная гипоспадия, Прадер 4, гонады в мошонке	Панель «НФП» — н/выявлено	c .G290C:p .G97A	новый
5	Промежностная гипоспадия, Прадер 3, гонады в брюшной полости	Панель «НФП» — н/выявлено	c .C133T:p .R45W	CM1616127 [15]
6	Промежностная гипоспадия, Прадер 3, гонады в паховых каналах	Панель «НФП» — н/выявлено	c .T62C:p .L21P	новый
7	Промежностная гипоспадия, Прадер 4, гонады в мошонке	Экзом — н/выявлено	c .A923C:p .K308T	новый

## ОБСУЖДЕНИЕ

HSD17B3 относится к большой группе 17β-гидроксистероиддегидрогеназ (HSD17B), регулирующих активность андрогенов и эстрогенов в различных органах и тканях организма. В настоящее время известно 14 форм HSD17B, большинство из которых были описаны в 1990-х [13]. За исключением HSD17B тип 5, все формы относятся к короткоцепочечным дегидрогеназам, семейства редуктаз (SDR). Несмотря на низкую гомологичность в строении, достигающую лишь 20–30%, функционально существует значительное перекрестное участие в реакциях между разными типами, особенно между HSD17B 1, 3, 5, 7 и 12 типов, позволяющее компенсировать дефициты друг друга внутри семейства. Между тем функция HSD17B3 является незаменимой в период внутриутробного развития у плода мужского пола, и ее нарушения ассоциированы с одним из вариантов НФП 46,XY.

При рождении наружные гениталии у пациентов 46,XY имеют строение ближе к феминному, поэтому такие дети как правило регистрируются в женском паспортном поле, и поводами для обращению к врачу являются обнаружение гонад в паховой области или вирилизация в период полового созревания [12][16], что было отмечено и нами (табл. 1).

Механизм поздней вирилизации до конца не ясен. Предполагается, что в пубертате на фоне повышения ЛГ в гонадах синтезируется значительное количество андростендиона, часть которого превращается в тестостерон. Ведущая роль здесь отводится ферменту HSD17B5, который, как и HSD17B3, способен конвертировать андростендион в тестостерон. Экспрессия HSD17B5 продемонстрирована как в экстрагонадной ткани [17], так и в клетках Лейдига [18], поэтому в период пубертата возможно несколько источников секреции тестостерона.

Гормональная диагностика заболевания основана на определении уровней андростендиона, тестостерона и соотношения между ними. При соотношении A/T выше 1,0 диагноз высоковероятен [19]. В детском возрасте соотношение A/T исследуется после проведения пробы с хорионическим гонадотропином, благодаря чему точность диагностики увеличивается до 90%. В обследованной нами группе с биаллельными мутациями данное соотношение у всех пациентов превышало 1.

По-видимому, первое описание пациента с дефицитом HSD17B3 было сделано Neher and Kant в 1965 г. [20], которые показали in vitro частичный дефицит фермента в ткани яичка у пациента с НФП 46,XY, что, однако, было расценено как проявление синдрома резистентности к андрогенам. Дефицит HSD17B3 как причина НФП 46,XY впервые документирован Saez et al., представившими данные гормонального обследования пациента с женским фенотипом при рождении, вирилизацией и увеличением грудных желез (гинекомастией) на фоне пубертата [16]. В 1994 г. Geissler et al. представили случаи молекулярно-генетического подтверждения дефицита HSD17B3, доказав связь заболевания с дефектами гена HSD17B3 [1].

К настоящему моменту описано не менее 70 различных мутаций в данном гене [21]. Среди нашей группы пациентов выявлена высокая частота встречаемости делеции c.729_735del:p .V243fs — на 9 хромосомах. Данный вариант впервые описан Andersson et al. у пациента из Польши [2], а в дальнейшем также найден при обследовании пациентов с НФП в Турции [22] и Египте [23]. Следующим по частоте изменением в обследованной нами группе была мутация в сайте сплайсинга интрона 3 c.277+4C>T. Данный вариант является частой находкой при дефиците HSD17B3, особенно среди жителей Европы [12][21].

Как уже отмечено выше, при дефиците HSD17B3 может быть ошибочно диагностирован СРА. В обследованной нами когорте пациентов с биаллельными мутациями это имело место в 4 случаях. Следует иметь в виду, что после удаления гонад без молекулярно-генетических исследований дифференцировать эти два состояния не представляется возможным. Об этом, например, свидетельствует публикация Phelan et al., обследовавших 36 взрослых женщин с НФП 46,XY, которые наблюдались по поводу неполной формы СРА, и выявивших мутации в гене HSD17B3 у 13 из 36 (36%), тогда как изменения в гене AR были обнаружены лишь в 1 случае [13].

Особое место в нашем исследовании занимают пациенты с моноаллельными вариантами в гене HSD17B3 (табл. 2). Фенотипически эти случаи отличаются от представленной выше группы пациентов с биаллельными мутациями. Как правило у них отмечалось смешанное строение наружных гениталий при рождении (Прадер 3–4), что явилось причиной регистрации большинства пациентов в мужском поле. В данной группе у 1 пациента была выявлена частая для дефицита HSD17B3 патогенная сплайсинг-мутация c.277+4A>T. У 3 пациентов обнаружен миссенс-вариант c.133C>T:p .R45W. Данный вариант был описан ранее при НФП 46,XY [15][24], между тем его клиническая значимость не является доказанной. Следует отметить, что, по референсным базам данных, средняя популяционная аллельная частота данного варианта сравнительно высокая (0,0017), однако он ни разу не обнаружен в гомозиготном состоянии [https://gnomad.broadinstitute.org/]. В целом, роль моноаллельных вариантов в гене HSD17B3 в патогенезе НФП 46,XY нельзя пока считать доказанной. Как и в отношении ряда других рецессивных заболеваний, высказываются предположения о существовании олигогенного механизма, когда сочетание мутаций в нескольких генах может оказывать аддитивный эффект и приводить к НФП [15][24].

## ЗАКЛЮЧЕНИЕ

В данной работе представлен опыт применения высокопроизводительного параллельного секвенирования в группе пациентов с НФП 46,XY. Оценен вклад дефицита HSD17B3 в структуру патологии, который составил 4,2% для биаллельных вариантов и 2,3% для моноаллельных изменений. В настоящем исследовании выявлено два часто встречающихся варианта в гене HSD17B3: c.729_735del:p .V243fs и c.277+4C>T. Полученные нами результаты демонстрируют важность ранней молекулярно-генетической верификации формы НФП с целью решения вопроса о дальнейшем ведении пациента с данной патологией.

## Ограничения исследования

Основным ограничением настоящего исследования является отсутствие данных гормонального профиля до момента верификации нуклеотидных изменений в гене HSD17B3 на 5 пациентов из 13, что могло привести к некоторому смещению результатов обследования, в частности соотношения А/Т.

## ДОПОЛНИТЕЛЬНАЯ ИНФОРМАЦИЯ

Источники финансирования. Работа выполнена при содействии Фонда поддержки и развития филантропии «КАФ».

Конфликт интересов. Авторы декларируют отсутствие явных и потенциальных конфликтов интересов, связанных с содержанием настоящей статьи.

Участие авторов. Все авторы одобрили финальную версию статьи перед публикацией, выразили согласие нести ответственность за все аспекты работы, подразумевающую надлежащее изучение и решение вопросов, связанных с точностью или добросовестностью любой части работы.
